# Factors associated with HIV and syphilis screenings among pregnant women at first antenatal visit in Lusaka, Zambia

**DOI:** 10.1186/s13104-020-05266-0

**Published:** 2020-09-07

**Authors:** Rindcy Davis, Xu Xiong, Fernando Althabe, John Lefante, Maria Luisa Cafferata, Musaku Mwenechanya, Friday Habulembe Mwanakalanga, Elwyn Chomba, Pierre Buekens

**Affiliations:** 1 Department of Epidemiology, Tulane University School of Public Health and Tropical Medicine, New Orleans, LA USA; 2 Department of Global Biostatistics and Data Science, Tulane University School of Public Health and Tropical Medicine, New Orleans, LA USA; 3 Institute for Clinical Effectiveness and Health Policy, Buenos Aires, Argentina; 4 University Teaching Hospital, Lusaka, Zambia; 5 New York, USA

**Keywords:** Mother-to-child transmission, Africa South of the Sahara, Zambia, HIV, Syphilis, Antenatal screenings

## Abstract

**Objectives:**

To identify characteristics associated with obtaining HIV and syphilis screenings of pregnant women attending a first antenatal visit in Lusaka, Zambia.

**Results:**

Among 18,231 participants from April 2015 to January 2016, 95% obtained HIV screening, 29% obtained syphilis screening, and 4% did not obtain antenatal HIV or syphilis screenings. Divorced/separated women were associated with a moderate decrease in prevalence of obtaining HIV (adjusted prevalence ratio (aPR) 0.88, 95% confidence interval (95% CI) 0.82, 0.95) and syphilis (aPR 0.51, 95% CI 0.27, 0.96) screenings compared to married women. Women with previous pregnancies were associated with a slight decrease in prevalence of obtaining HIV screening (aPR 0.97, 95% CI 0.95, 0.99) compared to women without previous pregnancy. Older women ≥ 35 years were associated with a slight decrease in prevalence of obtaining HIV screening (aPR 0.96, 95% CI 0.92, 0.99) compared to younger women. The statistically significant differences were not of clinical relevance as defined by a proportional difference of 10 percent. Findings of this study show that a vast majority of pregnant women are obtaining HIV screenings but not syphilis screenings during first antenatal visit. Provision of antenatal HIV and syphilis screening at first visit is only weakly related to patient level factors.

## Introduction

Despite advances in antenatal recommendations and treatment to prevent mother to child transmission (PMTCT) of HIV and syphilis, congenital infections represent a major global public health burden. The most effective method of prevention of mother to child transmission of HIV is to reduce maternal viral load through antiretroviral therapy (ART) during pregnancy and breastfeeding [1]. Without provision of antiretroviral treatment, the rate of vertical transmission of HIV can range from 15 to 45% [2, 3]. For prevention of congenital syphilis, one dose of benzathine penicillin G is effective treatment to prevent mother to child transmission of syphilis [4]. A single dose is sufficient to treat early syphilis, but syphilis of longer duration requires three doses to assure cure. Untreated syphilis can lead to fetal loss or stillbirth during pregnancy and surviving infants can be born preterm, low birth weight, or have active syphilis infection [5]. In addition, syphilis positive pregnant women are twice as likely to be HIV seropositive [6].

Antenatal screenings are a key step in prevention of HIV and syphilis transmission in order to identify seropositive women and start respective treatments [7, 8]. A focus on both HIV and syphilis screening during antenatal care has proven to be feasible, cost-effective, and prevent adverse birth outcomes [9–12]. Overall among women attending antenatal care in sub-Saharan Africa, approximately 60% of pregnant women received HIV testing and under 40% of pregnant women received syphilis testing [13, 14]. Specifically in Zambia, HIV screening of pregnant women was estimated to be 94% and syphilis screening was 85% during 2010–2013 [15].

Antenatal HIV and syphilis screenings are not universal in Zambia and greater sub-Saharan Africa [13, 16, 17]. Exploring individual level characteristics associated with antenatal HIV and syphilis screenings can better describe the current situation and assist with creating more targeted interventions to increase the provision of antenatal screenings. Previous studies have reported mixed findings on the association between women obtaining antenatal HIV and syphilis screenings during pregnancy and individual level characteristics such as age, adverse pregnancy outcomes, and marital status [18–22]. In addition, prior research focused on individual level factors associated with antenatal HIV or syphilis screenings have reported findings on either antenatal HIV or syphilis screenings. The objective of this present study was to identify clinically relevant individual factors associated with HIV and syphilis screenings at first antenatal visit in Lusaka, Zambia.

## Main text

### Methods

This is a cross-sectional study focusing on 13 Lusaka district clinics participating in the nine month baseline period of Preventing Congenital Syphilis (PCS), a facility-based, two-arm parallel cluster randomized implementation trial (ClinicalTrials.gov: NCT02353117; registered on February 2, 2015) [23]. The objective of the PCS implementation trial was to evaluate a behavioral multifaceted intervention to increase the use of evidence-based clinical procedures to increase syphilis screening and treatment during antenatal care in antenatal clinics in Kinshasa, Democratic Republic of the Congo and Lusaka, Zambia [24, 25].

The 13 antenatal clinics participating in the baseline period were selected based on geographic location within areas of Kafue and Chongwe located in the capital province of Lusaka. These health facilities that are designated as urban health centers with PMTCT programs in Lusaka District (91). During the baseline period, all women attending their first antenatal care visit at one of the 13 participating clinics during April 13, 2015 to January 10, 2016 were approached for study enrollment [24].

The inclusion criterion for this study included all consenting women who attended an antenatal care visit for the first time at one of the participating clinics in Lusaka, Zambia from April 2015 to January 2016 and recorded within the PCS baseline database. Women with completed data were included in the analyses. Antenatal care is defined as care during pregnancy before the initiation of labor. A first antenatal care visit is defined as the first visit at a participating health care facility during the current pregnancy. Exclusion criteria included women who did not/were unable to provide consent.

For enrolled participants, study personnel completed an antenatal care form at clinics [24]. Data was also collected from multiple source documents including antenatal care registry, antenatal card, PMTCT books, laboratory records, and nurses’ books [24]. Unavailable data from the source documents were collected from the mothers [24]. During the baseline data period, the study team produced detailed weekly and monthly data monitoring reports. Weekly calls and monitoring visits were made to continuously monitor study activities and data quality.

### Statistical analyses

The unit of data analysis is a pregnant woman. Data are summarized as count and percentage for all categorical variables. Log-binomial regression model with adjustment for cluster effects was used to estimate crude prevalence ratios (PR) with 95% confidence intervals (95% CI) and adjusted prevalence ratios (aPR) with 95% CI to determine which individual level factors were associated with the following dichotomous screening outcomes during first antenatal visit: (1) obtained HIV screening vs. no HIV screening (reference group (ref)) and (2) obtained syphilis screening vs. no syphilis screening (ref). Separate crude and adjusted PRs were calculated for variables pertaining to HIV and/or syphilis screenings among pregnant women with history of previous pregnancy using Poisson regression models with robust variance and adjustment for cluster effects. All aPRs and 95% CIs were adjusted for age, marital status, education, history of known syphilis infection, and previous pregnancy. These variables were selected a priori based on the existing literature [13, 26, 27]. All statistical analyses were conducted using SAS 9.4 (SAS Institute Inc., Cary, NC). Clinical relevance was defined by a proportional difference of 10 percent [28].

### Results

This study included 18,231 pregnant women. During first antenatal care visit, 95% of women obtained an HIV screening, 29% of women obtained a syphilis screening, and 4% of women did not obtain antenatal HIV or syphilis screening. Majority of the study participants were between 20 and 34 years of age (76%), married (88%), completed greater than primary school to less than or equal secondary school (55%), and had no history of syphilis infection (98%) (Table 1). Of the 13,396 women with previous pregnancy, 17% had a history of preterm birth, and 4% had a history of previous abortion and/or stillbirth (Table 1).

**Table 1:** Overall characteristics of pregnant women attending first antenatal care visit in Lusaka, Zambia, 2015–2016

Characteristics	n = 18,231	%
Screening status		
HIV screening only	12,340	67.7
Syphilis screening only	192	1.1
HIV screening and syphilis screening	5004	27.5
Neither syphilis or HIV	664	3.6
Age		
< 20	2456	13.5
20–34	13,792	75.7
≥ 35	1983	10.9
Marital status		
Married/with a partner	16,084	88.2
Single	2037	11.2
Widowed without current partner	55	0.3
Divorced/separated	55	0.3
Education		
≤ Primary complete	6625	36.3
Greater than primary complete to ≤ secondary school	9999	54.9
Greater than secondary school	1607	8.8
History of syphilis screening		
Yes	404	2.2
No	17,827	97.8
Number of previous pregnancies		
0	4835	26.5
1	4679	25.7
2	3671	20.1
3	2498	13.7
≥ 4	2548	14.0

Women with previous pregnancy	n = 13,300	%
Previous preterm		
0	12,790	96.2
1	431	3.2
≥ 2	79	0.6
Previous abortions and/or stillbirths		
0	11,122	83.6
1	1756	13.2
≥ 2	422	3.2

Majority of pregnant women had obtained HIV screening among all variable levels of age, marital status, education, history of syphilis infection, and previous pregnancy during first antenatal care visit in Lusaka, Zambia (Table 2). In multivariable regression analyses, pregnant women who were greater than or equal to 35 years of age (aPR = 0.96; 95% CI (0.92, 0.99)), divorced/separated (aPR = 0.88; 95% CI (0.82, 0.95)), had a history of syphilis infection (aPR 1.07, 95% CI 1.01, 1.15), and two or more previous pregnancies (aPR = 0.97; 95% CI (0.95–0.99)) were significantly associated with a decreased prevalence of obtaining an HIV screening.

**Table 2:** Individual-level characteristics and prevalence ratios for the outcome: pregnant women who obtained a HIV screening

Characteristics	HIV Screened, n = 17,374	HIV Screened, %	PR (95% CI)	aPR^a^ (95% CI)
Age				
< 20	2406	98.0	Ref	Ref
20–34	13,168	95.5	0.97 (0.96, 0.99)	0.99 (0.98, 1.00)
≥ 35	1800	90.8	0.93 (0.89, 0.96)	0.96 (0.92, 0.99)
Marital status				
Married/with a partner	15,312	95.2	Ref	Ref
Single	1972	96.8	1.02 (1.01, 1.03)	1.00 (0.99, 1.01)
Widowed without current partner	44	80.0	0.84 (0.65, 1.08)	0.86 (0.67, 1.09)
Divorced/separated	46	83.6	0.88 (0.82, 0.94)	0.88 (0.82, 0.95)
Education				
≤ Primary complete	6227	94.0	Ref	Ref
> Primary complete to ≤ Secondary school	9589	95.9	1.02 (1.01, 1.03)	1.01 (1.00, 1.02)
> Secondary school	1558	97.0	1.03 (1.01, 1.05)	1.02 (1.00, 1.04)
History of syphilis infection				
No	17,017	95.5	Ref	Ref
Yes	357	88.4	1.08 (1.01, 1.16)	1.07 (1.01, 1.15)
Previous pregnancy				
0	4733	97.9	Ref	Ref
1	4495	96.1	0.98 (0.97, 0.99)	0.99 (0.98, 1.00)
2	3461	94.3	0.96 (0.94, 0.98)	0.97 (0.95, 0.99)
3	2338	93.6	0.96 (0.93, 0.98)	0.97 (0.95, 0.99)
≥ 4	2347	92.1	0.94 (0.92, 0.97)	0.97 (0.95, 0.98)

Women with ≥ 1 previous pregnancy	n = 12,544	%	PR (95% CI)	aPR^a^ (95% CI)
Previous preterm births				
0	12,077	94.4	Ref	Ref
1	392	91.0	0.97 (0.94, 0.99)	0.97 (0.94, 1.00)
≥ 2	75	94.9	0.99 (0.93, 1.05)	1.00 (0.94, 1.06)
Previous abortions and/or stillbirths				
0	10,501	94.4	Ref	Ref
1	1653	94.1	0.99 (0.98, 1.01)	1.00 (0.98, 1.02)
≥ 2	390	92.4	0.97 (0.93, 1.01)	0.99 (0.95, 1.02)

^a^Adjusted prevalence ratios (aPR) and 95% confidence intervals (95% CI) were adjusted for age, marital status, education, history of known syphilis infection, and previous pregnancy

Majority of pregnant women did not obtain a syphilis screening at first antenatal visit among all variable levels of age, marital status, education, history of syphilis infection, and previous pregnancy during first antenatal care visit in Lusaka, Zambia (Table 3). Widowed women without a current partner were associated with an increase in prevalence of obtaining syphilis screening (aPR 1.49, 95% CI 1.08, 2.05) compared to married women. Conversely, divorced/separated women had a decrease in prevalence of obtaining syphilis screening (aPR 0.51, 95% CI 0.27, 0.96) compared to married women.

**Table 3:** Individual-level characteristics and prevalence ratios for the outcome: pregnant women who obtained a syphilis screening

Characteristics	Syphilis screened, n = 5196	Syphilis screened, %	PR (95% CI)	aPR^a^ (95% CI)
Age				
< 20	711	29.0	Ref	Ref
20–34	3940	28.6	0.99 (0.88, 1.11)	0.99 (0.88, 1.11)
≥35	545	27.5	0.95 (0.83, 1.09)	0.96 (0.83, 1.12)
Marital status				
Married/with a partner	4638	28.8	Ref	Ref
Single	527	25.9	0.90 (0.73, 1.10)	0.87 (0.71, 1.06)
Widowed without current partner	23	41.8	1.45 (1.05, 1.99)	1.49 (1.08, 2.05)
Divorced/separated	8	14.6	0.50 (0.27, 0.95)	0.51 (0.27, 0.96)
Education				
≤ Primary complete	1806	27.3	Ref	Ref
>Primary complete to ≤ secondary school	2971	29.7	1.09 (0.87, 1.37)	1.09 (0.87, 1.37)
> Secondary school	419	26.1	0.96 (0.67, 1.36)	0.96 (0.68, 1.36)
History of syphilis infection				
No	5091	28.6	Ref	Ref
Yes	105	26.0	1.10 (0.92, 1.31)	1.09 (0.93, 1.27)
Previous pregnancy				
0	1398	28.9	Ref	Ref
1	1342	28.7	0.99 (0.91, 1.08)	0.97 (0.89, 1.06)
2	1051	28.6	0.99 (0.91, 1.08)	0.97 (0.89, 1.06)
3	695	27.8	0.96 (0.84, 1.10)	0.95 (0.83, 1.09)
≥ 4	710	27.9	0.96 (0.84, 1.10)	0.97 (0.89, 1.06)

Women with ≥ 1 previous pregnancy	n = 3781	%	PR (95% CI)	aPR^a^ (95% CI)
Previous preterm births				
0	3610	28.2	Ref	Ref
1	143	33.2	0.98 (0.94, 1.02)	0.98 (0.94, 1.03)
≥ 2	28	35.4	1.02 (0.98, 1.06)	1.03 (0.98, 1.08)
Previous abortions and/or stillbirths				
0	3146	28.3	Ref	Ref
1	522	29.7	1.02 (1.00, 1.03)	1.02 (1.00, 1.03)
≥ 2	113	26.8	0.97 (0.93, 1.01)	0.97 (0.94, 1.01)

^a^Adjusted prevalence ratio (aPR) and 95% confidence intervals (95% CI) were adjusted for age, marital status, education, history of known syphilis infection, and previous pregnancy

### Discussion

Provision of screenings during the first antenatal care visit is essential to prevent vertical transmission of HIV and syphilis. However, antenatal HIV and syphilis screening rates vary dramatically across sub-Saharan Africa. Despite evidence demonstrating the cost-effectiveness and beneficial effects of prioritizing both antenatal HIV and syphilis screenings, previous studies have focused on individual level factors associated with either antenatal HIV or syphilis screenings. The current study is one of few that has investigated both types of antenatal screenings concurrently. Results of this study showed that only 29% of pregnant women obtained a syphilis screening compared to 95% of pregnant women who obtained HIV screening at first antenatal visit during the baseline period of the PCS implementation trial. Furthermore, the regression analysis showed limited clinically relevant individual level gaps in antenatal HIV and syphilis screenings among pregnant women attending a first visit. This is a positive finding in that provision of antenatal HIV and syphilis screenings by healthcare providers do not greatly differ by individual patient characteristics.

Education was the most common individual level factor assessed in previous studies. Multiple studies found that higher education among pregnant women was significantly associated with obtaining antenatal HIV and syphilis screenings [18–22]. The present study did not find a significant association between education level and screenings. Majority of participants in this study had received more than a primary education.

In the current study, the results showed a large gap between antenatal syphilis screenings and HIV screening at first visit which could not be attributed to individual level factors. The results of the PCS implementation trial showed that a multifaceted behavioral intervention with provision of supplies, including screening and treatment kits, can lead to more than 95% of pregnant women being screened and treated for syphilis without compromising HIV screening rates in Lusaka, Zambia and Kinshasa, Democratic Republic of the Congo [25]. Improvement of clinic-level factors demonstrated an increase in the provision of antenatal syphilis screenings. The findings of this study should be used to supplement the findings from the PCS implementation trial to inform future antenatal staff trainings, patient education, and future interventions that focus on scaling up antenatal HIV and syphilis screenings.

## Limitations

The findings of this study should be interpreted with consideration of study limitations. The cross-sectional design of this study prevents any causal interpretation of the significant associations. The variables available for this secondary analysis were limited and methods of data collection are not specifically designed for the purposes of this cross-sectional study. Although this study has a large sample size, because the health facilities were not randomly selected for the PCS baseline period, generalizability of the study findings should be limited to women attending first antenatal care visit at public clinics in Lusaka district. The findings of this study may also be subject to recall bias as the variables not recorded in clinic source documents are based on self-reports to trained study personnel without verification. Furthermore, the study is also susceptible to social desirability bias as the data collected by research staff reveal medical history and personal information on HIV and syphilis in pregnant women.

## Data Availability

The dataset supporting the conclusions of this article is available in the Mendeley Data repository titled, “Factors associated with HIV and syphilis screenings among pregnant women at first antenatal visit in Lusaka, Zambia.” https://data.mendeley.com/datasets/9b5x936n4g/draft?a=b2e6cf3e-8a33-40fe-bad1-8e6f3fedbb4c

## Abbreviations

HIV: Human Immunodeficiency Virus
ART: Antiretroviral therapy
PMTCT: Prevention of mother to child transmission
PCS: Preventing Congenital Syphilis
PR: Prevalence ratio
aPR: Adjusted prevalence ratio
